# Expression of Opioid Receptors in Cells of the Immune System

**DOI:** 10.3390/ijms22010315

**Published:** 2020-12-30

**Authors:** Jana Brejchova, Vladimir Holan, Petr Svoboda

**Affiliations:** 1 Laboratory of Biomathematics, Institute of Physiology of the Czech Academy of Sciences, 14220 Prague, Czech Republic; svobodap@fgu.cas.cz; 2 Department of Nanotoxicology and Molecular Epidemiology, Institute of Experimental Medicine of the Czech Academy of Sciences, 14220 Prague, Czech Republic; vladimir.holan@iem.cas.cz; 3 Department of Cell Biology, Faculty of Science, Charles University, 12843 Prague, Czech Republic

**Keywords:** opioid receptors, immune cells, stem cells, opioid drugs, addiction, chronic pain

## Abstract

The observation of the immunomodulatory effects of opioid drugs opened the discussion about possible mechanisms of action and led researchers to consider the presence of opioid receptors (OR) in cells of the immune system. To date, numerous studies analyzing the expression of OR subtypes in animal and human immune cells have been performed. Some of them confirmed the expression of OR at both the mRNA and protein level, while others did not detect the receptor mRNA either. Although this topic remains controversial, further studies are constantly being published. The most recent articles suggested that the expression level of OR in human peripheral blood lymphocytes could help to evaluate the success of methadone maintenance therapy in former opioid addicts, or could serve as a biomarker for chronic pain diagnosis. However, the applicability of these findings to clinical practice needs to be verified by further investigations.

## 1. Introduction

Natural and synthetic opioids represent the potent analgesics commonly used for the treatment of acute, chronic and inflammatory pain. Morphine is the prototypical opioid agonist to which all others are compared. Many patients, particularly those suffering from chronic pain, require long-term, high-dose analgesic therapy. However, for their additional qualities (positive emotional effect, even euphoria), opioids are also misused by healthy people, which leads to addiction, or even to intoxication. The clinical utility of morphine and other opioids is thus limited by undesired side effects, such as the development of analgesic tolerance, physical dependence (addiction), respiratory depression, constipation, and severe withdrawal symptoms manifesting after discontinuation of drug administration [1,2].

To replace the classical opioids for the treatment of pain and avoid the negative side effects, new drugs have been designed and synthesized. These included several morphine derivatives, and peptidomimetic analogues of enkephalins and endorphins. Although many of these substances were introduced into clinical practice as analgesics [3], only some of them (methadone, buprenorphine) have also been implemented for the treatment of addictive disorders. Methadone, a synthetic derivative of diphenyl heptane, synthesized in the 1960s, is an opioid analgesic used for the detoxification and substitution treatment of addiction to opioids, to slash craving and normalize physiological homeostasis (for review see Kreek [4]). Methadone is effective via oral consumption, and tolerance to and physical dependence on it develop more slowly than in the case of morphine. The symptoms of drug withdrawal after the abrupt discontinuation of methadone are milder, but are longer than those of morphine [5,6]. To date, methadone substitution therapy remains the preferable choice for the treatment of opioid addiction worldwide [7].

Optimistic expectations that the problems of tolerance, addiction and side effects of opioids will be solved remain unfulfilled [8]. Consequently, there is a need for a more safe medical treatment of addiction and withdrawal symptoms, and, most importantly, for the prevention of relapse. Since most approved medical treatments show only moderate effects on the chronic basis, a delineation of the proper biomarkers of addiction to opioid drugs is desirable.

## 2. Opioid Receptors and Biochemical Mechanisms of Homeostatic Adjustments to Chronic Opioid Stimulation

Opioid agonists such as morphine bind to and function through specific G protein-coupled receptors, the opioid receptors (OR). To date, four pharmacologically distinct types of OR have been identified, i.e., classical μ-, δ-, and κ-OR, and the non-classical receptor for nociceptin/orphanin FQ (NOP receptor) [9,10]. These receptors are localized in specific brain regions [11,12], the peripheral nervous system [12,13], the gastrointestinal tract [14], and can also be detected in some other cell types [15]. Agonist binding shifts the balance between active (R*) and non-active (R) forms of receptor towards the active conformation R*, which initiates intracellular signal transduction via the α and βγ subunits of the pertussis toxin-sensitive class of trimeric G proteins (G_i/o_). After activation, Gα and Gβγ dissociate from each other and regulate, directly or indirectly, adenylyl cyclases (AC), calcium and potassium ion channels, protein-kinases, phospholipases C, and mitogen-activated protein kinases [16,17,18,19]. The physiological significance of the signaling pathways initiated by OR lies in the modulation of the nociception, neuroendocrine and autonomic functions [20]. The opioid receptors also play an important role in reward and motivation [21,22,23], and affect emotional responses and cognition [22,24]. The involvement of the opioid receptors in pain control, drug abuse and mood disorders has been extensively studied and reviewed elsewhere [25,26,27,28,29,30].

The activation of OR normally results in the inhibition of AC activity, but the prolonged exposure of cultured cells or mammalian organisms to morphine was shown to induce the hyper-sensitization or super-activation of AC activity instead [31,32,33,34,35,36]. This effect was considered a biochemical basis for the development of opioid tolerance and dependence.

Our previous work on isolated plasma membranes (PM) from the forebrain cortex of rats exposed to increasing doses of morphine for 10 days indicated a desensitization of the G protein response to µ-OR (DAMGO) and δ-OR (DADLE) stimulation [37], and a specific increase in ACI (8×) and ACII (2.5×) isoforms [38]. The κ-OR (U-69593)-stimulated [^35^S]GTPγS binding and the expression level of ACIII-X in PM was unchanged. Behavioral tests of morphine-treated animals indicated that these animals were fully drug-dependent (opiate abstinence syndrome), and developed a tolerance to subsequent additions of drugs (analgesic tolerance; hot-plate and hind paw withdrawal tests) [37].

The increase in ACI and ACII was interpreted by us as a specific compensatory response to prolonged stimulation of the brain cortex OR by morphine. Importantly, the elevation of ACI and ACII was not detected in the membranes prepared from rats that received morphine for 10 days and were subsequently nurtured for 20 days in the absence of the drug. Thus, the marked increase in ACI and ACII faded away 20 days after the last dose of morphine.

The proteomic analysis of plasma membranes isolated from the forebrain cortex of rats treated with morphine for 10 days indicated the down-regulation of trimeric Gβ subunits (two-fold). Besides that, the up-regulation of proteins functionally related to oxidative stress and apoptotic cell death was noticed [39]. A subsequent study showed that, depending on the method used for protein detection and quantification, 28 (MALDI-TOF MS/MS) or 113 (MaxLFQ) proteins were identified as altered by chronic morphine. Importantly, in rats sacrificed 20 days after the last dose of morphine, the numbers of altered proteins decreased to 14 (MALDI-TOF MS/MS) and 19 (MaxLFQ), respectively [40]. We interpreted these results as the ability of living organism to (i) oppose the morphine-induced change in the target-tissue’s protein composition and (ii) elicit the partial return to the physiological norm after the complete withdrawal of the drug.

Although the phenomena of tolerance, dependence and withdrawal in the context of chronic opioid drug administration were intensively studied, it is still a major challenge to identify the neurobiological mechanisms that underlie the addiction. Unlike animals, neurobiological adaptations cannot be directly examined in humans. Therefore, there is a tendency to discover some peripheral markers that could help to evaluate the health status of human subjects chronically exposed to opioid drugs. In this context, one direction of research is represented by the studies oriented towards the analysis of the expression and function of OR in cells of the immune system.

## 3. The Multiplicity of Effects of Opioid Drugs on Cells of the Immune System

The first paper demonstrating the effects of opioids on the immune system was published in 1979 [41], and had a great impact on the following studies of OR in immune cells. The literature data published at the end of the last century mainly described the negative effects of opioids on the immune system [42,43]. These studies analyzed the effects of morphine and other opioids on individual cell populations and the functions of immune cells in vivo and in vitro. The reactivity of natural killer (NK) cells, macrophages, antibody-producing B cells and T cell subpopulations was characterized after the administration of opioids in mice, rats and humans. The majority of these studies showed the negative effects of opioids and the suppression of immune cell functions. The impact of opioids on NK cells was documented by their decreased cytotoxicity in morphine-treated mice [44]. The involvement of OR in this suppression was proven by the observation that µ-OR knock-out mice did not respond to morphine with a decreased NK cell activity [45]. The effects of opioids on NK cells were either direct or were mediated by signals through the neural system. Similarly, phagocytosis and other functions of macrophages were suppressed by morphine [46,47]. As in the case of innate immunity, the functions of cells of the adaptive immune system were also affected by opioids. However, the effects of opioids on the cells of adaptive immunity in morphine-treated animals were not always inhibitory, as was described for decreased antibody production [48] or impaired T cell functions [42,49]. Some publications showed that the influence of opioids on immune cells is more complicated [50,51]. With detailed knowledge of the immune system and after recognition of its individual cell populations, it has become apparent that the effects of opioids on some immune cell types could be suppressive, while the impacts of opioids on other immune cell populations and their functions are rather immunostimulatory. For example, we demonstrated that the production of pro-inflammatory cytokines (such as IL-2, IL-12) by spleen cells, or the secretion of NO by macrophages from mice treated with heroin, was significantly increased, while the production of anti-inflammatory cytokines (IL-4 and IL-10) was simultaneously rather suppressed [52]. As a consequence, skin allografts in heroin-treated mice were rejected more promptly than in control untreated or vehicle-treated recipients. Similarly, we showed the enhanced Concanavalin A (Con A)-induced proliferation of peripheral blood lymphocytes (PBL) isolated from heroin addicts in comparison with PBL from the control group of healthy donors [53]. In addition, the production of IL-2 and IFN-γ was higher in the group of heroin addicts than in the healthy controls. The enhanced proliferation of PBL, or the increased production of cytokines, observed in heroin addicts, were partially or completely normalized in the group of patients maintained on methadone [53]. On the other hand, the production of cytokines IL-1β, IL-6 and IL-8 was increased in the plasma of heroin addicts undergoing methadone replacement therapy [54]. The complexity of the effects of opioids on the immune system was supported by the data of Borner et al. [55], who showed that the treatment of human T lymphocytes with the opioids fentanyl, methadone, loperamide, and beta-endorphin resulted in a strong induction of IL-4 expression. In contrast, morphine and buprenorphine induced significantly lower levels of IL-4 mRNA. The changes in the expression of IL-4 suggest its possible role in the epigenetic modulation of μ-OR induction [56].

As reviewed by Liang et al. [57] and Eisenstein [58], the effect of OR ligands on cells of the immune system is not only immunosuppressive, as it was originally regarded, but is more complex. The summary of the up-to-date experience with treatment of acute and chronic pain caused by trauma, surgery or cancer indicates that the participation of OR in the function of the immune system is more complicated and unequivocal—ranging from immunosuppression on one side to immunostimulation on the other side.

## 4. Expression of OR in Cells of the Animal and Human Immune System

The direct effects of opioids on isolated immune cells in vitro exclude an indirect immunomodulation mediated by the nerve or neuroendocrine system, and suggest the presence of OR on leukocytes. The presence of OR on the surface of immunocompetent cells was proved for the first time by Carr et al. [59]. Since then, μ-, δ- and κ-OR have been found to be expressed in various immune cell types. These include animal and human immune cell-derived cell lines, as well as immune cells isolated from untreated animals and healthy human subjects (see Table 1).

Additionally, the inducible expression of OR in immune cells was reported. Transcripts of δ-OR were detected in the CD4+ T cells purified from murine splenocytes treated with mitogen Con A [60]. Increased expression induced by mitogen was reported also for κ-OR [61]. Recently, we described the inducible expression of δ- and µ-OR in rat spleen lymphocytes. The analysis of μ-, δ- and κ-OR content in these cells showed that the immunoblot signals of µ- and δ-OR proteins were undetectable in fresh/primary spleen lymphocytes; however, stimulation with Con A resulted in the up-regulation of µ- and δ-OR. The κ-OR were expressed already in primary cells, and their expression was increased 2.4-fold by Con A. The validity of immunoblot data was confirmed by flow cytometry. The stimulation of spleen cells with Con A caused a highly significant increase in the number of µ-, δ- and κ-OR-expressing cells—7.0-, 8.5- and 5.7-fold, respectively [62]. However, we observed this increase only in cells that were permeabilized prior to immunolabeling. In un-permeabilized lymphocytes, this effect of Con A stimulation was not detectable. Altogether, the flow cytometry results could be interpreted as evidence of the intracellular localization of the newly synthesized OR in Con A-stimulated cells. This provides an explanation of why the specific OR agonists DAMGO (μ-OR), DADLE (δ-OR) and U59693 (κ-OR) were unable to stimulate trimeric G proteins, and why membrane fractions prepared from Con A-stimulated cells did not exhibit the specific binding for the commonly used radioligands, [^3^H]diprenorphine and [^3^H]naloxone [62]. Our results were fully consistent with the literature’s data indicating that the ligand binding sites of OR in cells of the immune system differ from those present in the brain [63,64,65]. The Con A-induced increase in the expression of µ-, δ- and κ-OR was associated with a specific decrease in the expression levels of their cognate trimeric G proteins, G_i_1α/G_i_2α. The Gα and Gβ subunits belonging to other G protein families were unchanged. The level of β-arrestin-1/2 was also decreased by Con A. Thus, the down-stream regulatory proteins of the OR signaling cascades, i.e., G_i_1α/G_i_2α and β-arrestin-1/2, were expressed in high amounts already in un-stimulated spleen cells, and were significantly decreased by mitogen stimulation [62].

Besides mitogens, the expression of OR in cells of the immune system was shown to be induced by various other stimuli. Transcripts of δ-OR were detected in murine-purified T cells co-stimulated with monoclonal antibodies (mAbs) anti-CD3 and anti-CD28 [66]. In murine thymocytes, co-stimulation with CD3/CD28 mAb resulted in the expression of µ-OR [67]. Moreover, µ-OR expression was also found to be induced by the activation of thymocytes with various cytokines, such as IL-1β, IL-2, IL-7, IFNγ, TNFα, and TGFβ [67]. These cytokines are known to be present at functional levels in the thymus gland and to play a role in T cell development. The strongest up-regulation of µ-OR transcript levels was detected in thymocytes incubated with TGFβ. A substantial increase in µ-OR expression was also induced by IFNγ, IL-1β, and IL-2. On the other hand, a much weaker effect on µ-OR expression was exhibited by TNFα and IL-7 [67]. Overall, these results provided evidence that the expression of OR in immune cells is induced by their activation.

In contrast to the findings presented in Table 1, it was reported that human peripheral blood mononuclear cells (PBMC) isolated from healthy volunteers express NOP receptor mRNA, but do not express mRNA of either µ-, δ- or κ-OR [68]. The absence of µ-OR transcripts in primary unstimulated human T cells was also described by Borner et al. [69]. However, the µ-OR expression was found to be inducible by the stimulation of primary human T cells with IL-4 [69], or activation with CD3/CD28 mAb [70]. When compared to neuronal cells, the µ-OR expression level in immune cells was found to be 15 to 200 times lower [69]. In contrast to that, the NOP receptor expression levels in human immune cells were shown to be comparable to the levels detected in cells of the central nervous system [71].

Additionally, OR expression was found to be increased by morphine treatment. When CEMx174 cells (the human T/B hybrid cell line) were treated with morphine, µ- and κ-OR were significantly up-regulated, at the level of both mRNA and protein [72,73]. The question arises of whether the expression of OR in immune cells is modulated also under in vivo conditions by the long-term exposure of the human body to opioid drugs or pathological pain states. An overview of the studies that attempted to answer this question is presented in the following two sections.

## 5. Detection of OR in Cells of the Immune System of Drug-Addicted Humans

The immunological effect of opioid addiction was originally investigated by Mazzone et al. [96] in granulocytes isolated from chronic heroin abusers, long-term methadone-maintained former heroin users and age-matched healthy individuals. Only HIV seronegative subjects were enrolled in the study to avoid the confounding factor of HIV infection. One of the examined parameters was the expression of OR. For this purpose, polymorphic nucleocytes were labeled with fluorescent analogue of naloxone and analyzed by flow cytometry. Compared to the healthy controls, the number of OR was significantly higher in the granulocytes from both heroin users and methadone maintenance subjects. This increase was inversely correlated with the reduced chemotactic responsiveness of polymorphic nucleocytes. The increased expression of OR was also detected in the neutrophils, monocytes, and lymphocytes of former heroin addicts undergoing chronic treatment with naltrexone [97]. Again, only HIV seronegative subjects were examined.

In contrast to the abovementioned studies that strictly avoided the HIV-infected addicts, the work of Beck et al. [98] assessed the effect of HIV infection on the expression of opioid receptors on the peripheral white blood cells of intravenous drug users (IVDUs) undergoing methadone therapy. The analysis of lymphocytes, monocytes and granulocytes of HIV-positive and HIV-negative IVDUs treated with methadone was carried out by flow cytometry using polyclonal antibodies against human µ-, δ- and κ-OR. Much higher absolute numbers of OR-positive cells were detected in the HIV-negative IVDU group. The most striking difference was observed for δ-OR, which was markedly increased on the lymphocytes, monocytes and granulocytes of HIV-negative methadone-treated IVDUs when compared with HIV-positive methadone-treated subjects and healthy individuals. A substantial increase was also detected in the case of κ-OR in lymphocytes, whereas µ-OR levels were almost unchanged. The authors concluded that methadone treatment up-regulates OR expression in the immune cells of former IVDUs, and that this up-regulation is counteracted by HIV infection.

Contrarily, Toskulkao et al. [76] showed that the µ-OR and δ-OR mRNA levels in the PBL isolated from former heroin addicts on methadone substitution therapy were significantly lower (<50%) than in the lymphocytes collected from control, naive subjects. A corresponding result was provided by in vitro experiments. In cultured lymphocytes collected from naive human subjects, methadone significantly down-regulated both mRNA and protein levels of µ- and δ-OR. This effect was prevented by naloxone or by the pre-treatment of lymphocytes in culture with pertussis toxin. On the other hand, the up-regulation of one of the four known µ-OR splice variants (hMOR-1A) was described in PBL prepared from methadone-maintained subjects when compared with control subjects [99]. The authors hypothesized that the upregulation of hMOR-1A may serve as a feedback mechanism to restore the normal MOR membrane densities, which are lowered by methadone administration.

Recently, Shahkarami et al. [92] examined κ-OR and dynorphin mRNA levels in human PBL in relation to severe opioid use disorder (SOD). This study evaluated the blood samples of four different groups: subjects with SOD, subjects on methadone maintenance therapy, long-term (12 month) abstinent subjects with former SOD, and healthy controls. In contrast to the controls, κ-OR mRNA expression was significantly decreased in subjects with SOD and in methadone maintenance subjects. Unlike κ-OR, the expression of dynorphin peptide was markedly increased. The authors concluded that the down-regulation of κ-OR may be a compensatory response to the up-regulation of dynorphin that occurred after the chronic consumption of opioids. These changes seemed to be stable, as they were detected also in the abstinent subjects even after 12 months of abstinence. As such, the authors suggested that these long-term changes in the κ-OR and dynorphin expression levels in PBLs could serve as a biomarker of SOD development in the periphery.

## 6. Detection of OR in Cells of the Immune System of Chronic Pain Patients

Gunji et al. [93] analyzed the expression of κ-OR in peripheral blood cells and its relationship to the inflammatory activity and chronic pain in patients with rheumatoid arthritis. When compared to the healthy volunteers, κ-OR mRNA expression was markedly decreased in rheumatoid arthritis patients. This decrease was inversely related with the severity of symptoms. The lowest levels of κ-OR mRNA were found in patients with severe inflammatory changes and high pain scores. Regarding the expression pattern of κ-OR on peripheral blood cells, the mRNA was detected on T and B lymphocytes, and on the macrophages of both patients and healthy subjects. Moreover, κ-OR mRNA was found in the natural killer (NK) cells of rheumatoid arthritis patients. These results suggest the correlation of the expression levels of κ-OR mRNA and natural killer cell activity together with the anti-inflammatory effects and anti-nociception in rheumatoid arthritis. A corresponding result was also obtained from patients with osteoarthritis of the knee. The level of κ-OR mRNA in the peripheral blood mononuclear cells of patients was considerably lower than in healthy subjects, and was not affected by treatment with analgesics acetaminophen (paracetamol) or rofecoxib [100].

The report of Campana et al. [77] revealed that chronic pain had no effect on the expression of µ-OR in human PBMC. The levels of µ-OR mRNA in patients with chronic non-cancer pain were similar to those detected in healthy subjects. However, long-term treatment with intrathecal morphine or morphine plus local anesthetic bupivacaine induced a significant up-regulation of µ-OR mRNA. Moreover, the amount of µ-OR mRNA transcripts after 12 months of treatment was considerably higher in patients treated with morphine plus bupivacaine than in those treated with morphine alone. Elevated levels of µ-OR mRNA were confirmed in both groups of patients after another 12 months, i.e., after 24 month of treatment in total.

Most recently, Malafoglia et al. [101] presented a randomized clinical trial to find out whether the pre-surgical administration of opioids to osteoarthritic patients, enrolled for hip replacement, can prevent the onset of chronic pain and opioid tolerance or addiction development after surgery. Based on the hypothesis that the presence of OR on the surface of immune cells provides the strongest evidence of the correlation between pain and the activation of the immune system, the authors proposed lymphocyte OR as innovative biological markers of osteoarthritic pain.

The role of OR as biomarkers in patients with chronic pain was investigated by Raffaeli et al. [78]. This study analyzed the presence of µ-OR in the lymphocytes of patients with fibromyalgia and osteoarthritis, and in healthy subjects, representing the pain-free control group. Based on immunophenotyping analysis by flow cytometry, very low percentages (<2%) of T lymphocytes from all three groups of subjects were found to express µ-OR. In the case of B lymphocytes, considerably higher percentages (~20–44%) of cells expressing µ-OR were detected. The expression levels in both groups of patients were significantly lower than in the control group. Moreover, the percentage of µ-OR-positive B lymphocytes was inversely related to the intensity of pain. The amounts of µ-OR-positive B lymphocytes in moderate/severe pain fibromyalgia and moderate/severe pain osteoarthritis patients were comparable, and simultaneously they were significantly lower than in the control group or mild pain patients. The authors suggested that the determination of the percentage of µ-OR-positive B lymphocytes in patients suffering from chronic pain could be used as a biomarker in order to improve the objectivity of the diagnosis of chronic pain states.

Besides the immune cells of the peripheral blood, changes in µ-OR expression associated with the chronic pain state were observed in the immune cells of the gastrointestinal tract. The analysis of the colonic mucosa of patients with irritable bowel syndrome revealed significantly increased mRNA and protein levels of µ-OR when compared with the asymptomatic controls. In addition, the increased expression was also reported for β-endorphin, an endogenous ligand of µ-OR. Microscopic analysis identified the presence of µ-OR and β-endorphin immunoreactivities in CD4+, EMR-1+, and CD31+ cells, indicating the expression of µ-OR and β-endorphin by mucosal T-helper lymphocytes, eosinophils, and leukocytes, respectively. This result suggests the involvement of the opioid system in the immune-related compensatory role in visceral pain in irritable bowel syndrome patients [102]. 

In the case of the non-classical NOP receptor, its expression in peripheral blood cells was examined in the end-stage cancer patients suffering from chronic pain, and in septic patients. A significantly higher expression of NOP receptor mRNA was found in cancer and septic patients in comparison with healthy controls. On the other hand, a lower expression of pre-pronociceptin mRNA was found in both groups of patients than in control subjects. As the RNA for the analysis was isolated from the whole blood, the cell types expressing NOP receptor and pre-pronociceptin could not be identified. Although no association of mRNA levels with severity of pain was observed, there was some association with the inflammatory markers. These results suggested the role of the NOP-N/OFQ-system in inflammatory states [103]. Detailed information about the participation of NOP receptor activation in inflammatory diseases can be found in the review by Gavioli et al. [104]. The regulation of NOP receptor expression in response to the inflammatory stimulus was also studied in human PBL in order to identify the involved regulatory signaling pathways. The ERK and p38 were detected as the major signaling pathways regulating the expression of the NOP receptor under inflammatory conditions [94].

## 7. OR in Progenitor and Stem Cells; Inflammation and Injury 

The expression of OR was also demonstrated in various types of stem cells, which play an important role in the development of the organism, and in adulthood they participate in hematopoiesis and tissue regeneration and repair. For example, Steidl et al. [105] described the expression of G protein-coupled receptors of neuromediators in primary human CD34+ hematopoietic stem and progenitor cells. Similarly, Liu et al. [106] concluded that methionine enkephalin could be an effective inducer of dendritic cells derived from mouse bone marrow progenitors. All three types of OR have also been detected in embryonic stem cells, and in various progenitor and stem cells within the neural system [107,108,109]. We therefore studied the expression of OR in mouse and human mesenchymal stem cells (MSCs). These cells can be found in nearly all tissues in the body, where they contribute to tissue regeneration and immunological homeostasis. It has been shown that MSCs possess potent immunoregulatory and anti-apoptotic properties, produce numerous cytokines and growth factors and have the ability to migrate to the sites of inflammation or injury, where they inhibit the local inflammatory reactions and support the healing process [110,111]. Using immunoblot and flow cytometry analyses, we showed that human bone marrow-derived MSCs express μ-, δ- and κ-OR, and that this expression was enhanced in the presence of pro-inflammatory cytokines. In addition, morphine modified the MSC phenotype and altered the differentiation and secretory properties of these cells. While the expression of some immunoregulatory molecules, such as indoleamine-2,3-dioxygenase or cyclooxygenase-2, was increased in MSCs in the presence of morphine, the secretion of growth factors, such as hepatocyte growth factor or vascular endothelial growth factor, was inhibited by morphine [112]. Furthermore, we recently demonstrated that morphine decreases the expression of adhesive molecules on MSCs, and that the migration and organ distribution of exogenous MSCs systemically administered in syngeneic mice are altered in morphine-treated recipients [113]. In addition, the migration of therapeutically administered MSCs to the site of injury was significantly decreased in recipients treated acutely or chronically with morphine. All these observations indicate that the decreased and aberrant healing of damaged tissue observed after opioid administration [114,115,116] could be caused by the negative effect of opioids on stem cells, which are involved in healing and regenerative processes.

## 8. Conclusions

The optimistic expectations that the problems of tolerance, addiction and side effects of opioids will be solved remain unfulfilled. Consequently, there is a need for a safer medical treatment of addiction and withdrawal symptoms, and, most importantly, for the prevention of relapse. Since most approved medical treatments show only moderate effects on the chronic basis, the delineation of the proper biomarkers of addiction to opioid drugs is desirable.

Neurobiological adaptations cannot be directly examined in humans. Therefore, there is a need to discover some peripheral markers that could help to evaluate the health status of human subjects chronically exposed to opioid drugs. In this context, one direction of research is oriented towards the analysis of the expression and functional state of OR in the cells of the immune system.

The analysis of peripheral blood cells of drug-addicted subjects on methadone maintenance therapy provided evidence that the long-term exposure of the human body to opioid drugs modulates the expression of OR in the cells of the immune system. However, the results were equivocal, as increased as well as decreased expression levels of OR were detected.

Recent data support the view that the effects of OR ligands on cells of the immune system are not only immunosuppressive, as was originally thought, but are more complex. The summary of up-to-date experience with the treatment of acute and chronic pain caused by trauma, surgery or cancer indicates that the final outcome ranges from immunosuppression to immunostimulation.

The expression of OR in human immune cells was also shown to be modulated by pathological pain states, such as rheumatoid arthritis, osteoarthritis or fibromyalgia. In this case, more consistent results were obtained, as the receptor expression levels were inversely related to the pain intensity and the severity of symptoms.

Altogether, studies of OR in the cells of the immune system suggested that the OR in human immune cells may serve as possible biomarkers of opioid drug addiction in periphery, or as an advantageous diagnostic tool for the characterization of chronic pain states.

Further studies are needed to verify/delineate the applicability of these findings to clinical practice and to determine the role/involvement of the individual OR subtypes in these pathological situations in more details.

## Figures and Tables

**Table 1:** Expression of OR in immune cell-derived cell lines and in untreated immune cells isolated from animals and humans.

Opioid Receptor	Origin	Cell Type	References
µ-OR	Rat	Peritoneal macrophages	[74]
	Monkey	PBMC, polymorphonuclear cells	[75]
	Human	T/B hybrid cell line (CEMx174 cells)	[75]
		Polymorphonuclear cells, monocytes/macrophages, CD4+ T cells	[75]
		PBL	[76]
		PBMC	[77]
		T and B cells	[78]
		NK cells	[79]
δ-OR	Mouse	T and B cell lines	[80]
		Splenocytes	[81,82]
	Monkey	PBMC	[83]
	Human	T, B, and monocytic cell lines, T/B hybrid cell line (CEMx174 cells)	[80,83,84]
		PBL	[76]
		NK cells	[79]
κ-OR	Mouse	Thymoma and macrophage cell lines	[85,86]
		Splenocytes	[87,88]
		Thymocytes	[88,89,90]
	Rat	Spleen lymphocytes	[62]
	Monkey	PBMC	[91]
	Human	T and B cell lines, T/B hybrid cell line (CEMx174 cells)	[80,84,91]
		PBMC, CD4+ T cells	[91]
		PBL, monocytes	[80,92]
		T and B cells, macrophages	[93]
		NK cells	[79]
NOP receptor	Human	T, B, and monocytic cell lines	[71]
		PBL, monocytes	[71,94]
		Polymorphonuclear cells	[95]
		PBMC	[68]
		NK cells	[79]

## Abbreviations

AC		adenylyl cyclase
Con A		Concanavalin A
IVDU		intravenous drug users
MSCs		mesenchymal stem cells
OR		opioid receptors
PBL		peripheral blood lymphocytes
PBMC		peripheral blood mononuclear cells
SOD		severe opioid use disorder
